# Local Ischemia and Increased Expression of Vascular Endothelial Growth Factor Following Ocular Dissemination of *Mycobacterium tuberculosis*

**DOI:** 10.1371/journal.pone.0028383

**Published:** 2011-12-05

**Authors:** Seema M. Thayil, Thomas A. Albini, Hossein Nazari, Andrew A. Moshfeghi, Jean-Marie A. Parel, Narsing A. Rao, Petros C. Karakousis

**Affiliations:** 1 Department of Medicine, Johns Hopkins University School of Medicine, Baltimore, Maryland, United States of America; 2 Bascom Palmer Eye Institute, University of Miami, Miami, Florida, United States of America; 3 Doheny Eye Institute, University of Southern California, Los Angeles, California, United States of America; 4 Department of International Health, Johns Hopkins Bloomberg School of Public Health, Baltimore, Maryland, United States of America; Fundació Institut d'Investigació en Ciències de la Salut Germans Trias i Pujol. Universitat Autònoma de Barcelona. CIBERES, Spain

## Abstract

The pathogenesis of intraocular tuberculosis remains poorly understood partly due to the lack of adequate animal models that accurately simulate human disease. Using a recently developed model of ocular tuberculosis following aerosol infection of guinea pigs with *Mycobacterium tuberculosis*, we studied the microbiological, histological, and clinical features of intraocular tuberculosis infection. Viable tubercle bacilli were cultivated from all eyes by Day 56 after aerosol delivery of ∼200 bacilli to guinea pig lungs. Choroidal tuberculous granulomas showed reduced oxygen tension, as evidenced by staining with the hypoxia-specific probe pimonidazole, and expression of vascular endothelial growth factor (VEGF) was detected in the retinal pigment epithelium (RPE) and photoreceptors. Fundoscopic examination of *M. tuberculosis*-infected guinea pig eyes revealed altered vascular architecture and chorioretinal hemorrhage by Day 56 after infection. This model may be useful in further elucidating the pathogenesis of ocular tuberculosis, as well as in developing tools for diagnosis and assessment of antituberculosis treatment responses in the eye.

## Introduction

Tuberculosis (TB) remains a major global public health concern [1]. Many industrialized nations have noted an increase in the proportion of cases presenting with extrapulmonary TB in recent years [2]–[4]. In the USA, the proportion of extrapulmonary cases increased from 16% in 1993 to 21% in 2006 [5]. However, the pathogenesis of intraocular TB remains poorly understood partly due to the lack of adequate animal models that accurately simulate human disease. We recently developed a model of ocular TB resulting from hematogenous dissemination of *Mycobacterium tuberculosis* (*Mtb*) following aerosol delivery of the organisms to guinea pig lungs [6]. In this study, we used this model to begin to explore the mechanisms driving the pathophysiology of ocular TB. Vascular endothelial growth factor (VEGF) is known to induce retinal angiogenesis and exudation in numerous ocular diseases, including inflammatory cystoid macular edema [7] and experimental autoimmune uveitis [8]. VEGF is upregulated by inflammatory cytokines such as IL-6 [9], tumor necrosis factor-α (TNF-α) [10], and IL-8 [11], and may play a major role in inflammatory diseases such as rheumatoid arthritis [12], asthma [13], Churg-Strauss syndrome [14] and Behcet's disease [15]. In addition, granuloma formation in pulmonary sarcoidosis [16] and schistosomiasis [17] has been characterized by localized VEGF expression.

Animal models of pulmonary TB have demonstrated localized tissue hypoxia in necrotic granulomas [18]–[20]. Since VEGF expression is upregulated in response to hypoxia [21], we hypothesized that VEGF levels are increased at foci of TB infection. In support of this hypothesis, VEGF expression has been demonstrated in *Mtb*-infected human alveolar macrophages [22] and neurotuberculomas [23], [24]. The present study documents tissue hypoxia within choroidal granulomas and VEGF expression in the retinal pigment epithelium (RPE) and, to a lesser extent, the photoreceptors following ocular dissemination *of Mtb* in guinea pigs.

## Results

### Organ bacillary burden and evidence of hypoxia and VEGF expression in lungs

Guinea pigs were aerosol-infected with wild-*type Mtb* CDC1551, yielding an implanted inoculum of 2.2±0.11 log_10_ CFU/lung on the day after infection. The bacilli multiplied exponentially in the lungs during the first 14 days after infection and thereafter maintained a stable lung census for the duration of the study (Table 1). Guinea pig eyes did not become culture-positive until Day 28 after infection (3/4 eyes), yielding a mean bacillary burden of 1.9±1.1 log_10_ CFU. By Day 56 after infection, all animals had detectable CFU in the eyes, with an average CFU burden of 2.9±0.15 log_10_, which was maintained at a steady level until Day 84 after infection (Table 1).

**Table 1:** Comparison of mean log_10_ CFU/organ recovered from guinea pigs after aerosol infection with *Mtb* CDC1551.

Days after	Mean log_10_	Mean log_10_	Guinea pigs with
Infection	CFU/lung	CFU/eye	detectable CFU in eye
**1**	2.2±0.1	Nil	Nil
**14**	6.7±0.02	Nil	Nil
**28**	6.3±0.2	1.9±1.1	3/4 (75%)
**56**	6.8±0.8	2.9±0.2	4/4 (100%)
**84**	5.9±0.1	2.9±0.1	4/4 (100%)

**Figure 1:**
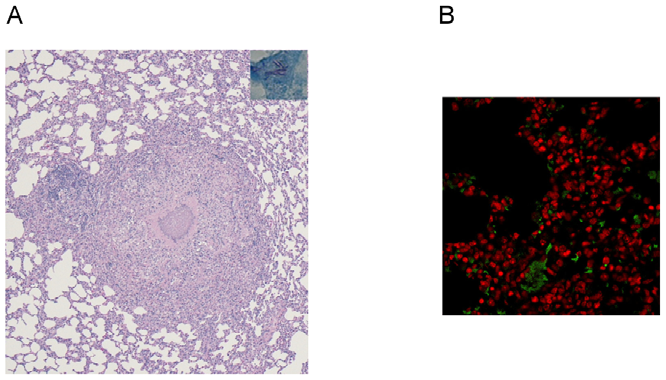
The lungs of *Mtb*-infected guinea pigs show evidence of tissue hypoxia and VEGF expression. A. Hematoxylin-eosin stain of guinea pig lungs on Day 56 after aerosol infection revealing well-circumscribed granuloma primarily comprising lymphocytes and epithelioid histiocytes with central necrosis (2×), containing acid-fast bacilli (inset; 60×). B. VEGF staining within TB granulomas in the lungs of guinea pigs infected with *Mtb* CDC1551 on Day 56 post-infection (40×).

Histological evaluation of the lungs at Day 56 after infection revealed well-circumscribed granulomas consisting primarily of lymphocytes and epithelioid histiocytes, with few plasma cells and central necrosis (Fig. 1A), and the presence of acid-fast bacilli (Fig. 1A inset). Lung sections were stained with the hypoxia-specific probe pimonidazole hydrochloride, revealing distinct regions of hypoxia in the lung granulomas by Day 56 after infection (data not shown). VEGF expression was also found to be prominent in areas surrounding these granulomatous lesions (Fig. 1B), but absent in the lungs of uninfected animals (data not shown).

### Evidence of tissue hypoxia in TB choroidal granulomas and VEGF staining in retinal pigment epithelium

**Figure 2:**
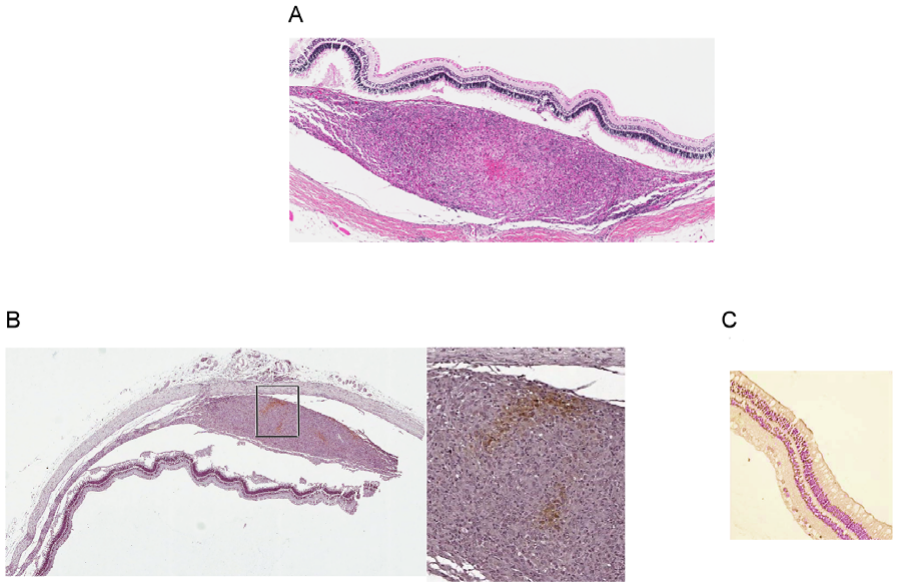
*Mtb*-infected guinea pig eyes show choroidal granulomas with tissue hypoxia. A. H&E section of *Mtb* CDC1551-infected guinea pig eye exhibits a typical choroidal granuloma with central necrosis at Day 56 after infection (7×). B. Pimonidazole HCl-stained section of eye from *Mtb* CDC1551-infected guinea pig shows areas of focal staining, indicating regions of hypoxia, in choroidal granuloma (2× and 10×). C. Choroidal tissue from uninfected control guinea pigs of the same age do not show staining with the hypoxia-specific probe pimonidazole (10×).

**Figure 3:**
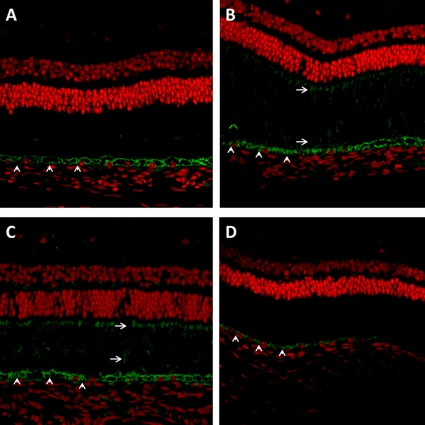
VEGF expression (seen in green) in guinea pig retinal pigmented epithelium (RPE) and photoreceptor outer segments as detected by immunohistochemistry (40×). Sections are from *Mtb*-infected eyes on Day 28 (A), Day 56 (B), and Day 84 (C) after aerosol infection and from uninfected control eyes (D). Photoreceptor outer segments (arrows) in panels B and C stain for VEGF; however, the photoreceptor layer in control eyes (D) and on Day 28 does not demonstrate VEGF expression. Retinal pigment epithelium (arrowhead) stains for VEGF at all time points.

Histological evaluation of the infected eyes showed prominent thickening of the choroid with granulomatous inflammation and central necrosis by Day 56 (Fig. 2A), as seen in cases of human ocular TB, but acid-fast organisms could not be detected within granulomas on multiple sections examined, likely due to the relatively small number of bacilli present in infected eyes (Table 1). At Day 56 and Day 84, choroidal granulomas stained positively for pimonidazole (Fig. 2B), indicating the presence of tissue hypoxia [25], [26], whereas uninfected ocular tissues showed no evidence of pimonidazole staining (Fig. 2C). VEGF staining was detected in the retinal pigment epithelium (RPE) of *Mtb*-infected eyes at Days 28, 56, and 84 after infection, but absent in uninfected controls (Fig. 3). VEGF expression was also observed by Day 56, and to a greater extent at Day 84 after infection, in the photoreceptor outer segments of all *Mtb*-infected eyes, but was absent in uninfected eyes.

### Retinal Imaging

**Figure 4:**
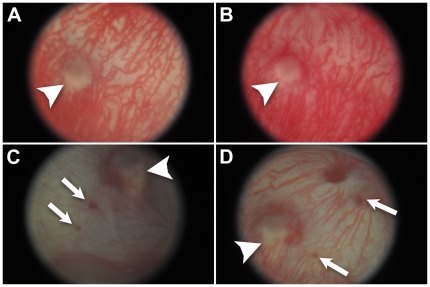
Chorioretinal hemorrhage and attenuated vasculature in the eyes of *Mtb*-infected guinea pigs. A and B. Fundus photographs of the posterior pole of the right eye in two uninfected guinea pigs. Arrow heads point to the optic nerve. Note pattern of choroidal circulation. C. Fundus photo of peripheral retina of the left eye of an infected guinea pig on Day 56. Arrows indicate chorioretinal hemorrhages. Note attenuation of choroidal vessels and creamy deep choroidal lesion with overlying vessels (arrow head); this lesion likely represents a choroidal granuloma. D. Posterior pole fundus photograph of left eye of an infected guinea pig on Day 56. An arrow head points to the optic nerve. Arrows point to hemorrhages. Note attenuated choroidal vasculature.

The retinal images of animals infected with *Mtb* showed decreased choroidal vasculature and areas of chorioretinal hemorrhage in three out of four animals at Day 56 after infection (Figs. 4C and 4D). All four animals exhibited evidence of hemorrhage by Day 84 after infection. In some animals on Day 56 and Day 84, deep choroidal creamy lesions were observed with overlying choroidal vasculature consistent with choroidal granuloma (Fig. 4C). The uninfected age-matched control animals did not show hemorrhage or other significant findings by retinal imaging at corresponding time points (Figs. 4A and 4B).

## Discussion

Extrapulmonary granuloma formation following aerosol infection of *Mtb* in animal models has not been well studied. This study provides microbiological evidence of ocular TB disease within 28 days after aerosol infection of guinea pigs, accompanied by the development of granulomas in a clinically relevant site that allows for direct observation by means of fundus photography. In guinea pigs, the retina is not vascularized and is oxygenated by the underlying choroidal circulation. Consequently, retinal vasculitis, which can be seen in clinical intraocular TB [27], [28], cannot be observed in the guinea pig. In addition, vitritis or cataract obscuring a view to the choroidal vasculature was not observed on fundus examination in the current study. These findings indicate that in the guinea pig model of intraocular TB, the inflammation remains predominantly confined to the choroid, consistent with the histopathological findings previously described in this model [6].

Immunohistochemical evaluation revealed the presence of hypoxia in TB choroidal granulomas, consistent with prior studies of TB granulomas in the lungs [19], [20], [29]. It is unlikely that choroidal hypoxia is a consequence of generalized hypoxia associated with pulmonary TB since pimonidazole staining is not observed in the choroid or other organs of *Mtb*-infected guinea pigs, including brain and spleen, in the absence of focal granulomas. Consistent with prior evidence of VEGF expression in human *Mtb*-infected lungs [22], we also detected increased expression of VEGF in the tissue surrounding lung TB granulomas in guinea pigs. T-lymphocytes upregulate VEGF expression *in vitro* as a response to MHC class II-mediated presentation of purified protein derivative of tuberculin [30] and *Mtb*-infected macrophages demonstrate a 6-fold upregulation of VEGF gene expression [31]. The current study demonstrates localized expression of VEGF associated with TB infection at an extrapulmonary site. VEGF expression was detected primarily in the RPE of *Mtb*-infected eyes. The RPE is known to be a source of VEGF and the maintenance of normal choroidal tissue is dependent on VEGF production by the RPE [32]. The associated finding of chorioretinal hemorrhage on fundus examination is consistent with increased vascular permeability and VEGF-mediated chorioretinopathy [33]. Moreover, VEGF expression appeared most intense in the photoreceptors by Day 84, suggesting increasing expression at this site over time. We hypothesize that VEGF could be upregulated in *Mtb*-infected lungs and RPE by at least two potential mechanisms: inflammatory mediators involved in *Mtb* infection may directly induce VEGF expression [34] and/or local inflammation may lead to vascular occlusion and hypoxia, resulting in VEGF upregulation [21]. Future studies will examine if there is a mechanistic link between tissue hypoxia and VEGF expression in the eye.

Previous research has focused on the use of VEGF as a biomarker for active TB disease. Although plasma VEGF levels did not correlate with disease activity in one study [31], other studies have suggested that plasma VEGF can be used as an indicator of active pulmonary TB [22], [35]. Greater VEGF levels have been documented in exudative pleural effusions due to TB as compared to transudates secondary to congestive heart failure [36]. On the other hand, malignant pleural [37], [38] and pericardial [39] effusions were found to have even higher levels of VEGF than TB-related effusions. Greater antigen-stimulated levels of VEGF in combination with other cytokines in the supernatant of interferon-γ release assays were found to accurately differentiate active disease from latent TB infection in one study [40]. Furthermore, increased VEGF levels in plasma and/or CSF were found to correlate with activity in neurotuberculosis [23], [24], [41]. However, it is unlikely that local (i.e., aqueous or vitreous) VEGF levels can serve as a biomarker of active intraocular TB since VEGF expression may be upregulated as a result of many different posterior segment inflammatory conditions [42], [43].

VEGF may also serve as a therapeutic target to alter the immune response and tissue damage due to *Mtb* infection. Steroid treatment has been shown to improve outcomes in TB affecting the central nervous system [44]. VEGF-mediated blood-brain barrier disruption is thought to exacerbate inflammation in TB meningitis [41] and *in vitro* induction of VEGF production in human monocytic THP-1 cells by *Mtb* sonicate or culture supernatant could be completely abrogated by corticosteroid treatment. Similarly, in a brain tumor model, dexamethasone was shown to inhibit VEGF production and thereby decrease the permeability of the blood-brain barrier [45]. Local anti-VEGF agents are commonly employed in the treatment of ocular disease by means of intravitreal injection [46], exhibiting sufficient choroidal penetration to inhibit choroidal neovascularization. Local inhibition of VEGF may similarly decrease the permeability of the blood retina barrier and favorably augment the inflammatory response in active ocular TB. On the other hand, inhibition of VEGF may decrease granuloma vascularity and promote caseation, which may increase tissue destruction [47].

The guinea pig model described in this study is highly relevant to studying the pathogenesis of ocular TB since involvement of the eye was observed after infection of animals physiologically, i.e., via aerosol. We believe the route of eye infection is hematogenous rather than through direct inoculation for several reasons. First, all lesions in the eye were present in the choroid, which is the typical location in cases of human ocular TB due to its rich vascularity and also because of the posterior location of this structure, which is surrounded by sclera. Infection is unlikely through the anterior structures of the eye, i.e., conjunctiva and cornea since there was no histologic evidence of inflammation at these sites. Moreover, such a route of infection would lead to iris and ciliary body inflammation and such pathology was absent in these animals. Notably, on careful histological analysis of guinea pig eyes from our previous studies (when the animals were infected in the same aerosol exposure system but the implantation dose was 100-fold higher) [6], we found no evidence of inflammation of the external structures of the eye (cornea, sclera) to suggest direct extension from the outside. Second, we could not culture bacilli from any of the eyes at Day 1 and Day 14 after infection in the current study, although the organisms grew by more than 4 log_10_ in the lungs during this 2-week interval. On the other hand, tubercle bacilli could be cultured from the eyes at Day 28 and beyond, coincident with the appearance of organisms in the spleen and other organs, representing hematogenous spread from the lungs. The inoculum used to infect guinea pigs in this study (∼200 bacilli) is likely significantly higher than that typically required to infect humans. Therefore, future studies will evaluate the ability of *Mtb* to disseminate to the eyes and other organs of guinea pigs following infection with a lower inoculum via aerosol.

In conclusion, the animal model described in the current study may be useful in further elucidating the pathogenesis of ocular TB, as well as in developing tools for diagnosis and assessment of antituberculosis treatment responses in the eye.

## Materials and Methods

### Ethics Statement

All procedures were performed according to protocols approved by the Institutional Animal Care and Use Committee at the Johns Hopkins University (protocol number GP09M68). All guinea pigs were maintained and bred under specific-pathogen-free conditions and fed water and chow ad libitum.

### Bacterial strains and growth conditions

The JHU standard reference strain of MTB CDC 1551 [48] used for animal infections was grown in Middlebrook 7H9 broth (Difco, Sparks MD) supplemented with 10% oleic acid-albumin-dextrose-catalase (OADC, Difco), glycerol, 0.05% Tween 80 at 37C on a roller. The *in vitro* growth of this strain was assessed by measuring the optical density at 600 nm.

### Animal infections

Twenty female outbred Hartley guinea pigs (250–300 g) were purchased from Charles River Labs (Wilmington, MA) and were infected in a Madison chamber aerosol generation device (College of Engineering Shops, University of Wisconsin, Madison, WI) calibrated to deliver approximately 100 bacilli of wild-type MTB CDC1551 into guinea pig lungs, as previously described [49]. Four guinea pigs from each group were euthanized at Days 1, 14, 28, 56, and 84 after infection. At necropsy, lungs and eyes were removed aseptically and examined. The right eye from each animal was homogenized for CFU enumeration and the contralateral eye was processed for histology. The lungs were homogenized in 10–20 ml of phosphate-buffered saline (PBS) using a Kinematica Polytron Homogenizer with a 12-mm generator (Brinkmann Instruments, Inc, Westbury, New York) within a BSL-III Glovebox Cabinet (Germfree Laboratories, Ormond Beach, Florida), as previously described [20]. Eyes were homogenized in 2–3 ml PBS using a glass homogenizer. Undiluted eye homogenates and serial tenfold dilution of lung homogenates were plated on 7H11 selective agar (BBL). Plates were incubated at 37°C and CFU were counted 4 weeks later. The left eye of each animal was paraffin-embedded and stained with Hematoxylin-eosin and Ziehl-Neelson acid-fast staining. Ocular and lung tissues for these experiments were obtained from animals included in a larger study evaluating an MTB recombinant mutant, the results of which will be published separately.

### Retinal Imaging

Funduscopic imaging was obtained using a compact, contact GRIN lens [50], [51], designed and constructed using standard optical and mechanical components: a Nikon D500 digital camera, an otoscope, a Karl Storz 611C Xenon light source, standard optical lenses, and mounts [52]. Animals were anesthetized using 400 ml/kg body weight of anesthetic constituting ketamine (100 mg/ml) and Xylazine (20 mg/ml) in normal saline. Proparacaine hydrochloride 0.5% was used for additional topical anesthesia. Each pupil was then dilated with 10% phenylephrine. A small amount of 2.5% methylcellulose gel was applied to the eye and the camera was placed in direct contact with the corneal surface. Fundus images were recorded at Day 1, 14, 28, 56 and 84 after aerosol infection.

### Immunohistochemistry

At predetermined time points, guinea pigs were injected with pimonidazole hydrochloride (Hypoxiprobe-1, HPI, MA) 4 hours prior to euthanasia. Immediately upon sacrifice, the lower left lobe of the lungs and the left eyes were placed in 10% paraformaldehyde for 24 hours before histological specimens were prepared. The paraffin-embedded sections of the eye were deparaffinized, hydrated and quenched in 3% hydrogen peroxide. Antigen retrieval was performed at 40°C by exposing to 0.01% Pronase for 40 minutes. Tissue sections were treated with IgG1 mouse monoclonal antibody (HPI, MA) as the primary antibody and stained with the Streptavidin-Biotin 2 system, horseradish peroxidase according to manufacturer's instructions (DAKO). The sections were mounted and viewed using a Nikon Eclipse 55i microscope.

To detect VEGF expression in retina, retinal pigment epithelium and choroid, paraffin-embedded sections from non-exposed control animals and infected animals were subjected to immunohistochemical evaluation. Five-micron thick sections were deparaffinized and subjected to antigen retrieval by covering the sections with 10 mM sodium citrate buffer/0.05% tween-20 (pH 6.0), and heating them for 30 seconds. Slides were then cooled to room temperature for 30 minutes, rinsed with PBS/0.05% Tween-20, and blocked with 5% bovine serum albumin for 30 minutes at room temperature. Sections were incubated for 1 hour at 37°C with mouse monoclonal anti–VEGF antibody (1∶100; Abcam, Cambridge, MA). This antibody is known to stain isoforms 121, 165, 165B, 183, 189, and 206. Sections were washed three times with PBS and then incubated in the dark for 1 hour at room temperature with fluorescein isothiocyanate (FITC) conjugated goat anti–mouse IgG (1∶200; Jackson Immuno Research laboratories, West Grove, PA). The sections were then washed with PBS, cover slips were mounted with mounting medium containing propidium iodide (Vector Laboratories, Burlingame, CA), and samples were viewed under a Zeiss LSM-510 laser scanning confocal microscope. Isotype control and primary antibody replaced by 1% bovine serum albumin were used as negative controls.
